# Altered Brain Activity in Depression of Parkinson’s Disease: A Meta-Analysis and Validation Study

**DOI:** 10.3389/fnagi.2022.806054

**Published:** 2022-03-23

**Authors:** Dongning Su, Yusha Cui, Zhu Liu, Huimin Chen, Jinping Fang, Huizi Ma, Junhong Zhou, Tao Feng

**Affiliations:** ^1^Department of Neurology, Beijing Tiantan Hospital, Capital Medical University, Beijing, China; ^2^China National Clinical Research Center for Neurological Diseases, Beijing, China; ^3^Beijing Rehabilitation Hospital of Capital Medical University, Beijing, China; ^4^Hinda and Arthur Marcus Institute for Aging Research, Hebrew SeniorLife, Roslindale, MA, United States; ^5^Harvard Medical School, Boston, MA, United States; ^6^Parkinson’s Disease Center, Beijing Institute for Brain Disorders, Capital Medical University, Beijing, China

**Keywords:** Parkinson’s disease, depression, functional magnetic resonance imaging, multiscale entropy, brain activities

## Abstract

**Background:**

The pathophysiology of depression in Parkinson’s disease (PD) is not fully understood. Studies based upon functional MRI (fMRI) showed the alterations in the blood-oxygen-level-dependent (BOLD) fluctuations in multiple brain regions pertaining to depression in PD. However, large variance was observed across previous studies. Therefore, we conducted a meta-analysis to quantitatively evaluate the results in previous publications and completed an independent regions-of-interests (ROIs)-based analysis using our own data to validate the results of the meta-analysis.

**Methods:**

We searched PubMed, Embase, and Web of Science to identify fMRI studies in PD patients with depression. Using signed differential mapping (SDM) method, we performed a voxel-based meta-analysis. Then, a validation study by using multiscale entropy (MSE) in 28 PD patients with depression and 25 PD patients without depression was conducted. The fMRI scan was completed in anti-depression-medication-off state. The ROIs of the MSE analysis were the regions identified by the meta-analysis.

**Results:**

A total of 126 PD patients with depression and 153 PD patients without depression were included in meta-analysis. It was observed that the resting-state activities within the posterior cingulate gyrus, supplementary motor area (SMA), and cerebellum were altered in depressed patients. Then, in the validation study, these regions were used as ROIs. PD patients with depression had significantly lower MSE of the BOLD fluctuations in these regions (posterior cingulate gyrus: *F* = 0.856, *p* = 0.049; SMA: *F* = 0.914, *p* = 0.039; cerebellum: *F* = 0.227, *p* = 0.043).

**Conclusion:**

Our study revealed that the altered BOLD activity in cingulate, SMA, and cerebellum of the brain were pertaining to depression in PD.

## Introduction

Parkinson’s disease (PD) is the second most important age-related neurodegenerative disorder in older adults. PD patients suffer from multiple non-motor symptoms together with the motor issues. One of the most common non-motor symptoms in PD is depression, which impairs both mental and physical function and diminishes a person’s quality of life (Aarsland et al., 2011; Skorvanek et al., 2015). Depression in PD is believed to be closely associated with pathological changes in neurotransmitter systems (Schapira et al., 2017). During the last decade, studies using positron emission tomography (PET) observed that dopamine transporter availability and noradrenergic innervation in the striatum and limbic brain regions were reduced in PD patients with depression compared to PD patients without depression (Burn et al., 2012; Vriend et al., 2014); and the alterations in dopamine transporter availability and noradrenergic innervation influence neural activity in cortical regions (Pa et al., 2013; Meyer et al., 2019). Depression is also characterized by deficits of excitatory glutamate neurons and inhibitory GABA interneurons (Duman et al., 2019). Evidence of associations between glutamate and/or GABA levels and fMRI signal was found recently (Delli Pizzi et al., 2020; Demartini et al., 2020; Kiemes et al., 2021). Therefore, characterization of the altered cortical neural activities in PD patients with depression is of great significance to help better understand the pathology underlying it, which can help optimize the design of therapeutic strategies and protocols for PD patients with depression.

Multiple studies have characterized resting-state brain activities in PD patients with depression by measuring the dynamics of functional magnetic resonance imaging (fMRI) blood oxygen level-dependent (BOLD) signals. Most results showed that alterations in PD patients with depression were predominantly in the prefrontal cortex and limbic system (Luo et al., 2014; Wang et al., 2020). However, the sample sizes of the studies were relatively small, and large variances in study protocol and data analytic techniques were observed between studies (Luo et al., 2014). For example, amplitude of low-frequency fluctuation (ALFF) is used to detect the regional intensity of spontaneous fluctuations in the BOLD signal (Cordes et al., 2001), Wen et al. (2013) and Hu et al. (2015) observed increased ALFF in the temporal lobe, while Luo et al. (2014) reported similar ALFF values in this area (Sheng et al., 2014; Wang et al., 2018). The regional homogeneity (ReHo) calculates the synchronization of low-frequency fluctuations between a given voxel with neighboring voxels (Zang et al., 2004), and the degree centrality (DC) value calculates the centrality of a node by adding the centrality of adjacent nodes (Wu et al., 2013). Sheng et al. (2014) and Wang et al. (2020) reported alterations in DC and ReHo in the lingual gyrus and supplementary motor area (SMA) in PD patients with depression; however, other studies did not find significant differences in the lingual gyrus and SMA between groups. These kinds of inconsistency across studies thus largely limit the understanding and characterization of the neural-physiologic mechanisms underlying depression in PD.

Here, we therefore performed a coordination-based meta-analysis with the goal of quantitatively and systematically examining the results from previous studies. To validate the results of meta-analysis, we performed a region-of-interest (ROI)-based study using fMRI data from 28 PD patients with depression and 25 PD patients without depression. The regions identified in our meta-analysis were used as ROIs of the analysis. The previous studies in our meta-analysis used various method of analysis, including ALFF, ReHo, and DC, which reflected the alteration of BOLD signals on a single scale. However, it is known that resting-state neural activities within the brain are regulated by multiple components across multiple scales of time, ranging from milliseconds (e.g., the time to transmit neural impulses) to hours or days (e.g., circadian rhythms). The multiscale dynamics of resting-state BOLD fluctuations are thus complex, which provides key information on neurophysiological regulations (Yang et al., 2015). Studies have emerged to characterize such complex dynamics by using multiscale entropy (MSE) and have demonstrated that resting-state BOLD complexity is closely associated with important functional performance (Liu et al., 2019; Zhou et al., 2020). Here, we also used MSE to characterize the complexity of BOLD fluctuation in each ROI. Our primary hypothesis is that the complexity of BOLD fluctuations within those ROIs would be lower in PD patients with depression as compared to PD patients without depression, confirming the findings in the meta-analysis.

## Materials and Methods

### Meta-Analysis

#### Search Strategies

We searched the literature in PubMed, Embase, and Web of Science using the following free-text terms: (“Parkinson’s disease” OR “PD”) AND (“fMRI” OR “functional magnetic resonance imaging” OR “BOLD” OR “blood oxygen level dependent” OR “ALFF” OR “amplitude of low frequency fluctuation” OR “ReHo” OR “regional homogeneity” OR “DC” OR “degree centrality” OR “functional connectivity” OR “FC”) AND (“depression” OR “depressed” OR “mood” OR “emotion” OR “emotional” OR “psychology” OR “psychological” OR “neuropsychological” OR “psychiatric” OR “neuropsychiatric”). Next, we examined the references of the included studies to identify additional eligible publications. The final search was completed in March 2021.

#### Study Selection

After the literature search, a total of 2,312 articles were retrieved. We first removed the duplicates from the search results. Then, full text reports were obtained and screened in detail. The inclusion criteria were: (1) resting state fMRI studies comparing a group of PD patients with depression with a sample of PD patients without depression; (2) studies using metrics for measuring local characteristics of resting state fMRI data (Such as ReHo, DC, or ALFF/fALFF) and based on the whole brain analysis; (3) studies reporting results with coordinates in Montreal Neurological Institute (MNI) or Talairach space. The exclusion criteria were as follows: (1) review articles, case reports and editorial letters; (2) conference proceedings without full report publication; (3) participates duplicate; (4) undefined PD patients with depression or not enough information provided to determine whether depression was present; (5) no resting state fMRI. For all the articles that no whole brain results were reported in the papers, we contact the corresponding authors but there was no response we can receive. Any disagreement between the two researchers was resolved by discussion or consulting a third specialist. The study selection process is presented in a PRISMA flowchart.

#### Data Extraction

Two researchers independently extracted data, and discrepancies were resolved in a consensus meeting. When no consensus was reached, a third specialist was consulted. From all eligible studies, we extracted the following information: first author, year of publication, sample size, MRI type, analysis method, statistical threshold, standard stereotactic space and patient characteristics [age, Unified Parkinson’s Disease Rating Scale (UPDRS) score, medication state, Hamilton Rating Scale for Depression (HRSD) score, and Mini-mental State Examination (MMSE) score et al.]. We also extracted peak coordinates and effect size measures of regions with a significant difference between the PD patients with depression and PD patients without depression.

#### Quality Assessment

We assessed the quality of fMRI studies by criteria derived from the guidelines for reporting an fMRI study described by Poldrack et al. (2008). These criteria were aimed at ensuring that detailed descriptions of the methods and results are included in fMRI studies. The criteria consisted of nine domains, and their specifications are provided as Supplementary Date in Supplementary Material. Each study domain was scored at 0.5 or 1 point, and the points of all the domains were totaled; studies that scored ≥7.5 were considered good, those that scored 4–7.5 were considered fair and those that scored ≤4 were considered poor quality. Two researchers performed the quality assessment independently. When the scores of assessments were different between these two reviewers, a third researcher was invited to join the discussion until all three reviewers agreed with the score.

#### Meta-Analysis

Our meta-analysis was conducted using signed differential mapping (SDM). SDM was a voxel-based meta-analysis that enables investigators to combine neuroimaging studies reporting peak coordinates. It uses peak coordinates to recreate a statistical parametric map of the effect size of the differences between PD patients with depression and PD patients without depression in each study and then performs a random-effects variance-weighted meta-analysis in each voxel (Wolters et al., 2019). In our meta-analysis, we used the default effect size version of signed differential mapping (ES-SDM) kernel size and thresholds (FWHM = 20 mm, voxel *p* = 0.005, peak height SDM-Z = 1, cluster extent = 10 voxels).

#### Robustness Analysis

To assess the robustness of the results, we first performed a heterogeneity analysis using a random effects model with Q statistics (*p* < 0.005) to determine whether there were significant unexplained variabilities between the study groups in the results. Then, we conducted jack-knife analysis (*p* < 0.005) by systematically repeating the meta-analyses after excluding one study at a time to test the replicability of the results in the meta-analysis.

### Regions-of-Interests-Based Functional Magnetic Resonance Imaging Study

#### Participants

Twenty-eight PD patients with depression and 25 age- and sex-matched PD patients without depression were recruited via the Department of Neurology, Beijing Tiantan Hospital completed this study. Patients with a diagnosis of PD from three neurologists according to the 2015 Movement Disorder Society (MDS) criteria (Postuma et al., 2015) were included in both groups. Participants in both groups were excluded using the following criteria: moderate to severe head tremor, functional motor disorder (Tinazzi et al., 2021a,b), cerebrovascular disorders, antiparkinsonian treatment with dopamine agonists, antidepressant treatment or other psychiatric therapy, and cognitive impairment as defined by an MMSE score <24. For the PD patients with depression, depression was diagnosed based on the Diagnostic and Statistical Manual of Mental Disorders, Fifth Edition (DSM-V) criteria by an experienced, board-certified psychiatrist trained to administer structured clinical interviews. This study was approved by the Medical Ethical Review Committee of Beijing Tiantan Hospital. All procedures conformed to the Declaration of Helsinki. Every subject signed informed consent forms prior to participation.

#### Data Acquisition

The severity of clinical symptoms was assessed according to the Hoehn and Yahr (H&Y) rating scale (Hoehn and Yahr, 1967), and the motor part of the UPDRS (Hamilton, 1960). H&Y score was calculated in anti-Parkinsonian medication OFF state. The severity of depression symptoms was assessed according to the HRSD (Hamilton, 1960). The demographic and clinical data of the included participants are shown in Table 1.

**TABLE 1:** Demographic and clinical characteristics of all subjects.

	PD patients with depression	PD patients without no depression	*P*-value
Gender(female/total)	17/28	9/25	-
Age	63.61 ± 8.32	63.56 ± 8.79	0.984
Disease duration	8.19 ± 4.92	7.48 ± 4.68	0.600
MMSE	25.13 ± 3.66	26.54 ± 3.12	0.155
HRSD	15.79 ± 6.42	3.08 ± 1.96	<0.01
H&Y stage	2.64 ± 0.80	2.23 ± 0.67	0.063
UPDRS-III	39.93 ± 13.81	41.33 ± 13.70	0.776

*Values are represented as the mean ± standard deviation (SD). P-values were obtained using two sample t-test (P < 0.05). MMSE, Mini-mental state examination; HRSD, Hamilton Rating Scale for Depression; HAMA, Hamilton Anxiety Scale; H&Y stage, Hoehn and Yahr stage; UPDRS-III, Unified Parkinson’s disease rating scale part III.*

All the fMRI were completed in resting state, that is, no task was performed by the participants during the MRI scan. Patients underwent resting state fMRI after an overnight withdrawal of anti-Parkinsonian medication. Images were acquired on 3 T SIEMENS MAGNETOM Prisma scanner (Siemens Healthineers, Erlangen, Germany) using a 64-channel head coil. Structural 3D T1-weighted images were acquired with magnetization-prepared rapid gradient echo (MP-RAGE) sequence with the following parameters: repetition time = 2,300 ms, echo time = 2.26 ms, inversion time = 900 ms, flip angle = 8°, field of view = 256 mm × 256 mm, matrix = 256 × 256, number of slices = 186, voxel size = 1 mm × 1 mm × 1 mm with no gap. Resting state data were acquired using an echo-planar imaging sequence with repetition time = 750 ms, echo time = 30 ms, number of slices = 64, flip angle = 54°, field of view = 222 mm × 222 mm, matrix size = 74 × 74, voxel size = 3 mm × 3 mm × 3 mm with no gap. Resting state scans were carried out in a scanning run of 6 min and 11 s. Prior to scanning, foam padding and headphones were placed on the subjects to limit head motion and reduce scanner noise, respectively; also, patients were instructed to keep their eyes closed, relax but not fall asleep, and move as little as possible during scanning. Criteria for head motion correction are included as Supplementary Notes 1 in Supplementary Material.

#### Functional Magnetic Resonance Imaging Data Analysis

FMRI data were preprocessed using Resting-State fMRI Data Analysis Toolkit V1.24^1^ (RESTplus V1.24) (Jia et al., 2019). Data were first transformed into the NIFTI format, and the first 10 volumes were excluded for magnetization stabilization. The following steps were then performed: slice-time correction, motion correction, spatial normalization using the EPI MNI template, 8-mm kernel smoothing, and scaling to a percentage change from the mean. Data were then bandpass filtered to no less than 0.01 Hz to reduce low-frequency drifts and entered into a general linear model to remove the effects of 24 degrees of motion and their derivatives, nuisance cerebrospinal fluid (CSF), white matter, and global signal. No data were excluded because of excessive head motion.

The residual time series from this deconvolution was then used to calculate the MSE within each ROI. The ROIs were defined according to the results of our meta-analysis. Then, the MSE was used to quantify the complexity of each BOLD time series by calculating the entropies across five temporal scales. Greater averaged entropies reflected greater complexity. Specific calculation of MSE values and validation of the MSE results are included as Supplementary Notes 2 in Supplementary Material (Costa et al., 2002, 2005; Yang et al., 2013; Lindquist et al., 2019).

#### Statistical Analysis

Statistical analysis was performed with SPSS version 25 software. Means, standard deviations (S.Ds.) and percentages of selected descriptive characteristics were calculated for the study sample. Independent two-sample t-tests were used to examine the differences in demographic and clinical characteristics and MSE values in each ROI between the PD patients with depression and PD patients without depression. The significance level was set to *p* < 0.05 for all analyses.

## Results

### Meta-Analysis

#### Study Selection and Quality Assessment

**FIGURE 1:**
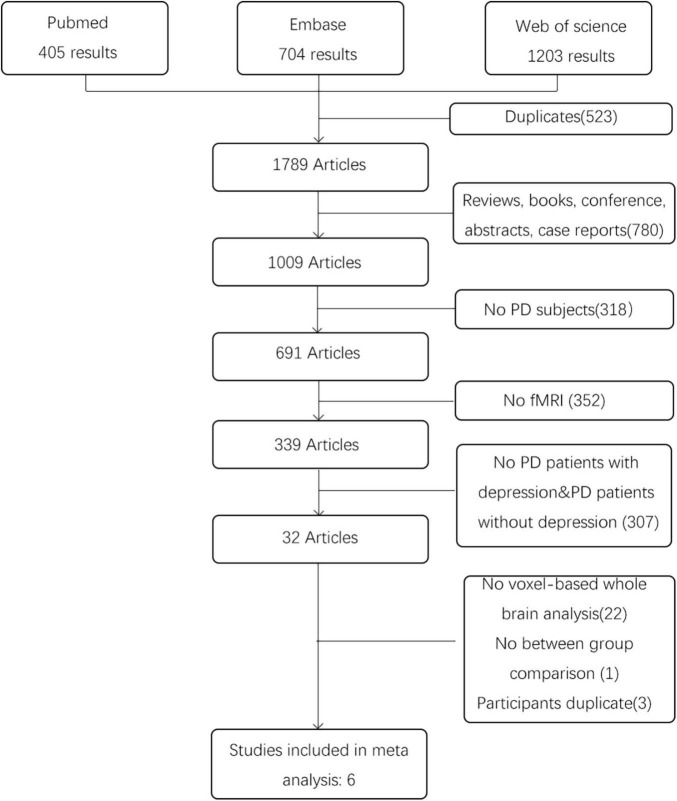
Study selection flow chart, performed according to the PRISMA 2020 guidelines.

According to the search strategy, we found 2,312 results, and 1,789 articles remained after removing duplicates. Then, we excluded 1,783 articles on the basis of our inclusion and exclusion criteria. The details are shown in Figure 1. Finally, six studies involving 126 PD patients with depression and 153 PD patients without depression were included in the meta-analysis. In these studies, age and sex were matched in two groups. The demographic and clinical characteristics including UPDRS, HRDS, and MMSE as well as imaging information of the included and excluded studies are provided in Table 2 and Supplementary Table 1.

**TABLE 2:** Demographic characteristics and experimental design of the included studies.

References		Hu et al., 2015	Luo et al., 2014	Sheng et al., 2014	Wang et al., 2018	Wang et al., 2020	Wen et al., 2013
**Sample size**	**Depression**	20	29	20	27	13	17
	**No depression**	39	30	21	27	20	16
**Gender (male/female)**	**Depression**	9/11	14/15	13/8	17/10	6/7	7/10
	**No depression**	26/13	15/15	13/7	19/8	11/9	8/8
**Disease duration**	**Depression**	5.35 ± 2.82	1.98 ± 1.64	3.4 ± 1.7	-	29.0 ± 22.4	6.4 ± 5.4
	**No depression**	6.50 ± 3.84	2.12 ± 1.30	4.0 ± 2.4	-	28.35 ± 1.98	5.6 ± 7.4
	***P***	0.217	0.72	0.224	-	0.994	-
**Age**	**Depression**	58.05 ± 7.72	51.5 ± 8.2	55.9 ± 7.4	60.8 ± 9.53	63.9 ± 10.6	64.4 ± 13.4
	**No depression**	54.6 ± 1.05	53.6 ± 10	53.7 ± 6.1	59.3 ± 12.6	58.4 ± 7.0	60.7 ± 18.7
	***P***	0.305	0.59	0.25	0.94	0.246	0.105
**MMSE**	**Depression**	-	27.4 ± 2.5	26.9 ± 1.7	26.4 ± 1.2	22.1 ± 4.5	29.5 ± 0.5
	**No depression**	-	27.0 ± 2.7	27.6 ± 2.0	27.4 ± 1.4	27.3 ± 2.7	29.2 ± 2.2
	***P***	-	0.52	0.096	0.001	<0.001	0.495
**UPDRS-III** (Skorvanek et al., 2015)	**Depression**	27.7 ± 1.32	28.3 ± 16.9	39.4 ± 10.8	17.0 ± 3.8	50.4 ± 8.4	42 ± 46
	**No depression**	28.2 ± 13.2	26.8 ± 12.4	43.8 ± 8.2	16.3 ± 3.8	43.1 ± 5.0	33.8 ± 24.2
	***P***	0.879	0.70	0.078	0.50	0.004	0.110
**HSD**	**Depression**	15.0 ± 4.8	18.31 ± 6.14	19.3 ± 5.0	13.3 ± 4.63	30.0 ± 3.5	15.2 ± 7.8
	**No depression**	6.8 ± 3.1	4.4 ± 2.73	6.4 ± 2.1	2.5 ± 1.8	3.1 ± 1.7	4.4 ± 4.4
	***P***	0.0001	<0.01	<0.01	<0.001	<0.01	<0.01
**Medication** ON/OFF		ON	OFF	OFF	OFF	NR	OFF
**Antidepressant medication**		NO	NO	NO	NO	NR	NO
**Criteria for**		DSM-V	DSM-IV	DSM-IV	DSM-IV	-	DSM-IV
**Scaner**		3T	3T	3T	3T	3T	3T
**Analysis**		ALFF	ALFF	ReHo	DC	ReHo	ALFF
**Software**		SPM8	SPM8	SPM8	GRETNA	DPARSE	SPM8
**Reported space**		MNI	MNI	MNI	MNI	MNI	MNI
**Minimum cluster size (voxel)**		34	50	68	13	16	16
**Corrected method**		AlphaSim	AlphaSim	AlphaSim	AlphaSim	FEW	AlphaSim

*Values are represented as the mean ± standard deviation (SD). P-values were obtained using two sample t-test (P < 0.05). Gender = male/female. MMSE, Mini-mental state examination; UPDRS-III, Unified Parkinson’s disease rating scale part III; HRSD, Hamilton Rating Scale for Depression; FEW, Family-wise error correction; DSM-IV, Diagnostic and Statistical Manual of Mental Disorders version four; DSM-V, Diagnostic and Statistical Manual of Mental Disorders version five; ALFF, Amplitude of low frequency fluctuation; ReHo, Regional homogeneity; DC, Degree centrality; MNI, Montreal Neurological Institute; DPARSF, Data Processing Assistant for Resting-State fMRI; SPM, Statistical Parametric Mapping software; NR, Not reported.*

During the quality assessment, three studies in our meta-analysis were classified as good, and the other three studies were deemed fair. The most common reasons for a deduction from the score were (1) the authors did not describe the inclusion or exclusion criteria of the study, (2) the authors did not specify how regions of interest were determined, (3) the authors did not describe detailed quality control measures, and (4) no slice coordinates were given for the figures. However, all of the studies in our meta-analysis scored above six, which meant that their quality reached an acceptable level. The specification of the quality assessment is presented Supplementary Date in Supplementary Material. We described the experimental design of the included studies in Table 2.

#### Differences of Neural Activity Between the Parkinson’s Disease Patients With Depression and Parkinson’s Disease Patients Without Depression

**FIGURE 2:**
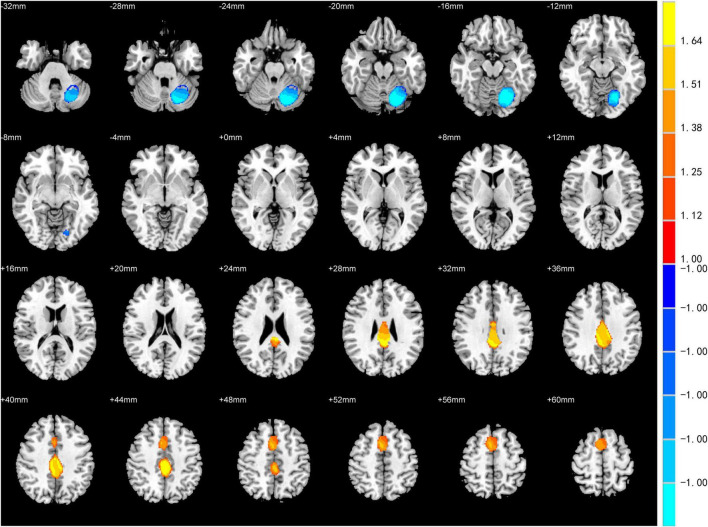
Statistically significant effects of meta-analysis for Parkinson’s disease (PD) patients with depression vs. PD patients without depression. Voxel threshold *p* < 0.005, peak height threshold: peak SDM-Z > 1.000, extent threshold: cluster size ≥ 10 voxels. Increased activity in PD patients with depression is indicated in yellow and decreased activity in blue.

The meta-analysis for differences between activation in the PD patients with depression and PD patients without depression yielded significant convergence of activation in the left posterior cingulate gyrus (peak MNI: *X* = 0, *Y* = –42, *Z* = 26; voxels = 1,966) and right SMA (peak MNI: *X* = 6, *Y* = 8, *Z* = 52; voxels = 1,008) (PD patients with depression > PD patients without depression). On the other hand, a significant difference was also found in the left cerebellar hemispheric lobule (peak MNI: *X* = –14, *Y* = –68, *Z* = –16; voxels = 2,011) (PD patients with depression <PD patients without depression) (Table 3 and Figure 2).

**TABLE 3:** Clusters of voxels with significant intergroup activation differences.

Neural region	Side	MNI coordinates			Voxels	*P*-value	SDM-Z
		*X*	*Y*	*Z*			
**Depression > No depression**							
Posterior cingulate gyrus	Left	0	–42	26	1966	0.00016	1.770
Supplementary motor area, BA 6	Right	6	8	52	1008	0.00076	1.557
**No depression > Depression**							
Left cerebellum, hemispheric lobule VI, BA 18	Left	–14	–68	–16	2011	0.00005	–1.601

*Voxel threshold p < 0.005, peak height threshold: peak SDM-Z > 1.000, extent threshold: cluster size ≥ 10 voxels. BA, Brodmann area; MNI, Montreal Neurological Institute.*

#### Robustness Analysis

Heterogeneity analysis from SDM showed that there was noteworthy between-study heterogeneity in Brodmann area (BA) 23, the SMA (BA 46), the middle frontal gyrus (BA 46), the cerebellum hemispheric lobule-VI (BA 19), the inferior frontal gyrus–orbital part (BA 47), the corpus callosum and the inferior frontal gyrus–opercular part (Supplementary Table 2). Jackknife sensitivity analysis revealed that abnormal activity in the cingulate gyrus was the most robust result. Other regions, including the SMA and cerebellum, were not robust in the meta-analysis, as they showed poor replicability of the peak coordinate (Supplementary Table 3). Since the number of included studies was less than 10, Egger’s test would not provide reliable results and was not performed.

### Regions-of-Interests-Based Functional Magnetic Resonance Imaging Study

The demographic and clinical characteristics of the 53 participants (28 PD patients with depression and 25 PD patients without depression) are presented in Table 1. No significant differences in these characteristics were observed between the two groups. Based on the results of the meta-analysis, three clusters were identified as ROIs: the cingulate gyrus (peak MNI: *X* = 0, *Y* = –42, *Z* = 26; voxels = 1,966), SMA (peak MNI: *X* = 6, *Y* = 8, *Z* = 52; voxels = 1,008), and cerebellum (peak MNI: *X* = –14, *Y* = –68, *Z* = –16; voxels = 2,011). We observed that compared to the PD patients without depression, in the group of PD patients with depression, MSE of BOLD decreased significantly in the posterior cingulate gyrus (No depression vs. Depression = 1.16 ± 0.06 vs. 1.12 ± 0.07, *F* = 0.856, *p* = 0.045), SMA (No depression vs. Depression = 1.13 ± 0.05 vs. 1.10 ± 0.05, *F* = 0.914, *p* = 0.039), and cerebellum (No depression vs. Depression = 1.08 ± 0.06 vs. 1.04 ± 0.05, *F* = 0.227, *p* = 0.043) (Supplementary Figure 1), which was consistent with the results of previous meta-analyses. Except ROIs-based analysis, we also performed a whole-brain analysis, however, no positive result was found after false discovery rate (FDR) correction.

## Discussion

Our study aims to characterize the alterations in neural activity of brain regions in PD patients with depression. Using a coordination-based meta-analysis of publications in this field, we provide first-of-its-kind evidence that PD patients with depression had the most consistent abnormalities in the posterior cingulate gyrus, SMA, and cerebellum. Then, to provide an independent validation of the meta-analysis results, we conducted an ROI-based analysis to measure the multiscale dynamics of these ROIs in PD patients with depression. All the patients didn’t take antidepressant medication. The results showed that compared to that in PD patients without depression, the complexity in ROIs was significantly lower in PD patients with depression, confirming the results of the meta-analysis that spontaneous activity of the posterior cingulate gyrus, SMA, and cerebellum pertain to the pathogenesis of depression in PD; these areas may serve as targets for the management and therapeutic strategies of PD patients with depression.

We observed alterations in resting-state neural activities within the cingulate gyrus, which is consistent with previous studies (Cardoso et al., 2009; Wen et al., 2013; Sheng et al., 2014). The cingulate gyrus is a key region of the prefrontal cortex (PFC)-limbic circuit, which has been linked to depression in both non-PD and PD cohorts (Cardoso et al., 2009; Luo et al., 2014). Neuroimaging evidence suggests that functional connectivity (FC) in this circuit is negatively correlated with depression severity (Du et al., 2017; Pantazatos et al., 2020), and increased FC between the PFC and cingulate gyrus is associated with depression-related gene orthodenticle homeobox 2 (OTX2) (Gärtner et al., 2019). Taylor and Liberzon (2007) for example, showed a potential pathological mechanism in which the inhibitory effects of the PFC on the limbic system, an important function in the regulation of mood, were impaired in people with depression. Taken together, the results here provided confirmatory evidence that alteration of the cingulate gyrus is a contributor to the pathophysiologic changes of PD patients with depression.

Similarly, alterations in the activities of the cerebellum were also observed in PD patients with depression. The cerebellum may participate in the processing of depression in PD via the cerebellar-cerebral circuit, which is formed by separate cerebellar subregions connected to distinct cerebral regions (D’Angelo and Casali, 2012). Dynamic FC in cerebellar-cerebral circuits decreased in depression patients, characterized by decreased connections of the cerebellar subregions with the default-mode, executive and affective-limbic networks (Alalade et al., 2011; Zhu et al., 2020). Decreased cerebellar-cerebral FC was proven to be correlated with the severity of depressive symptoms in PD patients, and it increased after electroconvulsive therapy (ECT) (Wei et al., 2020). Moreover, Ma et al. (2013) demonstrated that this circuit could serve as a biomarker to distinguish depression patients from HCs, suggesting the important role of this circuit in depression. All these results support the important role of the cerebellum in the pathogenesis of depression in PD.

Furthermore, we found that PD patients with depression had abnormal neural activity in the SMA. The SMA is located in the medial posterior third of the superior frontal gyrus and is mainly concerned with the motor symptoms of PD, such as posture and gait (Shirota et al., 2013). Recently, converging evidence has suggested that the SMA also plays a critical role in the integration of affective and cognitive functions (Nachev et al., 2008). A PET study revealed that treatment-resistant depression (TRD) patients were characterized by hypometabolism in the SMA (Li et al., 2015), and the symptoms of TRD patients were alleviated after the activity of SMA increased (Chen et al., 2018). However, the related studies are limited (Wang et al., 2018, 2020). Therefore, the mechanism by which SMA is involved in PD patients with depression still needs further research.

Of interest, all three regions indicated in our study showed altered function in depression patients as well (Liu et al., 2010; Bürger et al., 2017). Given the similarities between PD patients with depression and depression, most treatment strategies are the same. However, there are still some differences among treatment strategies. For example, the dopamine-releasing agent methylphenidate was not able to improve the depressive symptoms of PD patients due to degeneration of the dopaminergic innervation of the limbic system (Remy et al., 2005). In addition, most PD patients do not tolerate antipsychotics well because the dopamine-blocking action of these drugs contributes to PD symptoms (Ryan et al., 2019). For treatment-refractory depression, ECT leads to more adverse effects in PD patients than in purely depressed patients (Borisovskaya et al., 2016). Thus, for the better treatment of PD patients with depression, it is of significance for us to further study its pathophysiologic basis.

## Limitations

Our study has several limitations. First, the meta-analysis was limited by the small number of studies and a relatively high level of heterogeneity in study characteristics. To get more data to support our study, we contacted all the corresponding authors of studies without whole brain results in papers. However, there was no response we can receive. As shown in Table 2, different statistical or analysis methods, software packages and threshold settings were used in the included studies, which may influence our meta-analysis results. Therefore, our meta-analysis results must be interpreted cautiously. It is necessary to perform further subgroup analysis for studies with different measures, such as ALFF, ReHo, and DC. Unfortunately, due to the limited number of included articles, further subgroup analysis was not able to be performed. We suggest the validation of our findings through future studies consisting of an independent, methodologically homogeneous data set. Second, our study put together studies in which PD patients underwent fMRI in anti-Parkinsonian medication ON state and anti-Parkinsonian medication OFF state, this might be a potential confound on resting state fMRI signal. A subgroup analysis is needed to explore the effect of L-dopa on resting state fMRI signal. Third, the sample size of the PD patients with depression and PD patients without depression in our ROI-based fMRI study was relatively small. Therefore, fMRI studies with larger sample sizes are needed to confirm our results of ROI-based analysis. Fourth, we performed the whole-brain analysis but no significant results were observed by passing FDR correction. This may be due to the relatively small sample size of participants. We totally agree that the whole-brain analysis in participants with much larger sample size will help confirm and expand the findings of the current ROI analysis, which are warranted in future studies. Finally, limited by the number of studies, we did not perform a meta-analysis on functional connectivity studies of PD patients with depression. Future studies are expected to provide a better understanding of the functional connectivity networks involved in PD patients with depression.

## Conclusion

In conclusion, our meta-analysis revealed specific resting-state fMRI signal alterations in the cingulate gyrus, SMA and cerebellum in PD patients with depression. Our ROI-based fMRI analysis provided primary evidence that the complexity of neural activity was altered in the cingulate gyrus, SMA, and cerebellum. These findings are helpful in unraveling pathophysiology of depression in PD, and they also have the potential to serve as neuroimage biomarkers as well as provide novel targets for neuromodulation treatment for PD patients with depression.

## Data Availability Statement

The original contributions presented in the study are included in the article/Supplementary Material, further inquiries can be directed to the corresponding authors.

## Ethics Statement

The studies involving human participants were reviewed and approved by the Medical Ethical Review Committee of Beijing Tiantan Hospital. Number: KY 2018-080-02. The patients/participants provided their written informed consent to participate in this study.

## Author Contributions

DS contributed to the conception and design of the study. YC and DS contributed to the organization and execution of the research project and drafted the text, and prepared the figures. DS, YC, HC, JF, and HM contributed to acquisition, post-processing, and analysis of the data. JZ and TF revised the manuscript. All authors contributed to the article and approved the submitted version.

## Conflict of Interest

The authors declare that the research was conducted in the absence of any commercial or financial relationships that could be construed as a potential conflict of interest.

## Publisher’s Note

All claims expressed in this article are solely those of the authors and do not necessarily represent those of their affiliated organizations, or those of the publisher, the editors and the reviewers. Any product that may be evaluated in this article, or claim that may be made by its manufacturer, is not guaranteed or endorsed by the publisher.

## Footnotes

1
http://www.restfmri.net/forum/sites/default/files/200902_1511_RESTplus_v1.24.tar.gz
