# Supplementary figures and images for: Altered Brain Activity in Depression of Parkinson’s Disease: A Meta-Analysis and Validation Study
Source: Front Aging Neurosci. 2022 Mar 23;14:806054. doi: 10.3389/fnagi.2022.806054 (PMC8984499; doi:10.3389/fnagi.2022.806054)

**Supplementary Figure 1.** Violin plot of each individual sample MSE (multiscale entropy) value.

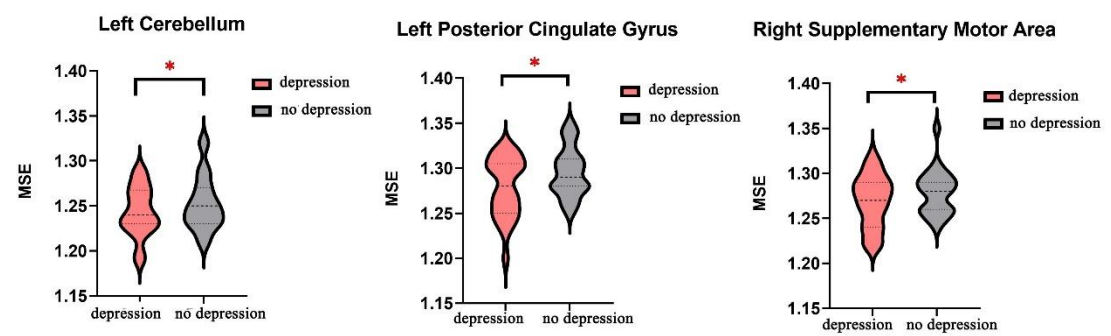

Supplement: Supplementary file 1 [file Image_1.pdf]
