# Supplementary material for: Altered Brain Activity in Depression of Parkinson’s Disease: A Meta-Analysis and Validation Study
Source: Front Aging Neurosci. 2022 Mar 23;14:806054. doi: 10.3389/fnagi.2022.806054 (PMC8984499; doi:10.3389/fnagi.2022.806054)
Supplement: Supplementary file 2 [file Data_Sheet_1.docx]

**Supplementary Date：Quality assessment**

**Figure1**: Quality assessment

Quality assessment derived from the guidelines of Poldrack et al. An overall score of ≥7.5 was considered as good (green), 4-7.5 as fair (orange) and ≤4 as poor (red) quality.

| Table 1. Luo et al (2013) | + | +/- | - | Other (CD, NR,NA)* |
| --- | --- | --- | --- | --- |
| 1. Did they give a full description of the study participants? |  | √ |  |  |
| 2. Did they give a full description of the psychological task used in fMRI? | √ |  |  |  |
| 3. Did they specify the spatial normalization procedure, including the atlas or template which is used to match the images to? | √ |  |  |  |
| 4. Did they specify how the regions of interest were determined? | √ |  |  |  |
| 5. Did they provide enough detail to reproduce the analysis? | √ |  |  |  |
| 6. Are all the empirical claims supported by a specific statistical test? | √ |  |  |  |
| 7. Did they describe and account for the multiple testing problem? | √ |  |  |  |
| 8. Do the figures and tables stand on their own? |  | √ |  |  |
| 9. Are the quality control measures documented? | √ |  |  |  |
| Quality Rating (Good, Fair or Poor) (See guidance) | 8 (Good) | | | |
| *Additional Comments (If POOR, please state why):*  *1：Inclusion criteria not clearly described*  *8: No slice coordinates given for figure1, figure2.* | | | | |
| *CD, cannot determine: NA, not applicable: NR, not reported | | | | |

| Table 2. wen et al (2013) | + | +/- | - | Other (CD, NR,NA)* |
| --- | --- | --- | --- | --- |
| 1. Did they give a full description of the study participants? |  | √ |  |  |
| 2. Did they give a full description of the psychological task used in fMRI? | √ |  |  |  |
| 3. Did they specify the spatial normalization procedure, including the atlas or template which is used to match the images to? | √ |  |  |  |
| 4. Did they specify how the regions of interest were determined? |  |  |  | NA |
| 5. Did they provide enough detail to reproduce the analysis? | √ |  |  |  |
| 6. Are all the empirical claims supported by a specific statistical test? | √ |  |  |  |
| 7. Did they describe and account for the multiple testing problem? | √ |  |  |  |
| 8. Do the figures and tables stand on their own? | √ |  |  |  |
| 9. Are the quality control measures documented? |  | √ |  |  |
| Quality Rating (Good, Fair or Poor) (See guidance) | 7 (Fair) | | | |
| *Additional Comments (If POOR, please state why):*  *1：Inclusion and exclusion criteria not clearly described*  4: This study doesn’t apply ROI based analysis or seed-based analysis.  *9: No covariates described for the applied mask.* | | | | |
| *CD, cannot determine: NA, not applicable: NR, not reported | | | | |

| Table 3. Hu et al (2015) | + | +/- | - | Other (CD, NR,NA)* |
| --- | --- | --- | --- | --- |
| 1. Did they give a full description of the study participants? | √ |  |  |  |
| 2. Did they give a full description of the psychological task used in fMRI? | √ |  |  |  |
| 3. Did they specify the spatial normalization procedure, including the atlas or template which is used to match the images to? | √ |  |  |  |
| 4. Did they specify how the regions of interest were determined? |  | √ |  |  |
| 5. Did they provide enough detail to reproduce the analysis? | √ |  |  |  |
| 6. Are all the empirical claims supported by a specific statistical test? | √ |  |  |  |
| 7. Did they describe and account for the multiple testing problem? | √ |  |  |  |
| 8. Do the figures and tables stand on their own? |  | √ |  |  |
| 9. Are the quality control measures documented? |  | √ |  |  |
| Quality Rating (Good, Fair or Poor) (See guidance) | 7.5 (Good) | | | |
| *Additional Comments (If POOR, please state why):*  4：Don‘t describe how the signal is extracted within ROI.  *8: No slice coordinates given for figure1, figure2.*  *9: No covariates described for the applied mask.* | | | | |
| *CD, cannot determine: NA, not applicable: NR, not reported | | | | |

| Table 4. wang et al (2018) | + | +/- | - | Other (CD, NR,NA)* |
| --- | --- | --- | --- | --- |
| 1. Did they give a full description of the study participants? | √ |  |  |  |
| 2. Did they give a full description of the psychological task used in fMRI? |  |  | √ |  |
| 3. Did they specify the spatial normalization procedure, including the atlas or template which is used to match the images to? |  | √ |  |  |
| 4. Did they specify how the regions of interest were determined? |  | √ |  |  |
| 5. Did they provide enough detail to reproduce the analysis? | √ |  |  |  |
| 6. Are all the empirical claims supported by a specific statistical test? | √ |  |  |  |
| 7. Did they describe and account for the multiple testing problem? | √ |  |  |  |
| 8. Do the figures and tables stand on their own? | √ |  |  |  |
| 9. Are the quality control measures documented? |  | √ |  |  |
| Quality Rating (Good, Fair or Poor) (See guidance) | 6.5 (Fair) | | | |
| *Additional Comments (If POOR, please state why):*  *2：No instructions for the resting-state functional measurement are described.*  3：*Atlas or template not specified*  4：Don‘t describe how the signal is extracted within ROI.  *9：No motion correction described.* | | | | |
| *CD, cannot determine: NA, not applicable: NR, not reported | | | | |

| Table 5. wang et al (2020) | + | +/- | - | Other (CD, NR,NA)* |
| --- | --- | --- | --- | --- |
| 1. Did they give a full description of the study participants? | √ |  |  |  |
| 2. Did they give a full description of the psychological task used in fMRI? |  |  | √ |  |
| 3. Did they specify the spatial normalization procedure, including the atlas or template which is used to match the images to? | √ |  |  |  |
| 4. Did they specify how the regions of interest were determined? |  |  |  | NA |
| 5. Did they provide enough detail to reproduce the analysis? | √ |  |  |  |
| 6. Are all the empirical claims supported by a specific statistical test? | √ |  |  |  |
| 7. Did they describe and account for the multiple testing problem? | √ |  |  |  |
| 8. Do the figures and tables stand on their own? |  | √ |  |  |
| 9. Are the quality control measures documented? |  | √ |  |  |
| Quality Rating (Good, Fair or Poor) (See guidance) | 6 (Fair) | | | |
| *Additional Comments (If POOR, please state why):*  *2：No instructions for the resting-state functional measurement are described.*  4: This study doesn’t apply ROI based analysis or seed based analysis.  8: *No slice coordinates given for figure1, figure2, figure3.*  *9：No motion correction described.* | | | | |
| *CD, cannot determine: NA, not applicable: NR, not reported | | | | |

| Table 6. Sheng et al (2014) | + | +/- | - | Other (CD, NR,NA)* |
| --- | --- | --- | --- | --- |
| 1. Did they give a full description of the study participants? | √ |  |  |  |
| 2. Did they give a full description of the psychological task used in fMRI? | √ |  |  |  |
| 3. Did they specify the spatial normalization procedure, including the atlas or template which is used to match the images to? | √ |  |  |  |
| 4. Did they specify how the regions of interest were determined? |  | √ |  |  |
| 5. Did they provide enough detail to reproduce the analysis? | √ |  |  |  |
| 6. Are all the empirical claims supported by a specific statistical test? | √ |  |  |  |
| 7. Did they describe and account for the multiple testing problem? | √ |  |  |  |
| 8. Do the figures and tables stand on their own? | √ |  |  |  |
| 9. Are the quality control measures documented? |  | √ |  |  |
| Quality Rating (Good, Fair or Poor) (See guidance) | 8.5 (Good) | | | |
| *Additional Comments (If POOR, please state why):*  *9: No covariates described for the applied mask.* | | | | |
| *CD, cannot determine: NA, not applicable: NR, not reported | | | | |
