# Supplementary material for: Altered Brain Activity in Depression of Parkinson’s Disease: A Meta-Analysis and Validation Study
Source: Front Aging Neurosci. 2022 Mar 23;14:806054. doi: 10.3389/fnagi.2022.806054 (PMC8984499; doi:10.3389/fnagi.2022.806054)
Supplement: Supplementary file 3 [file Data_Sheet_2.docx]

**Supplementary Table 1:** Demographic characteristics and experimental design of the excluded fMRI studies.

| Studies | Sample size | | Age^1^ | | MMSE^1^ | | Medication ON/OFF | UPDRS-III^1^ | | BDI^#^/HRSD* 1 | | LEDD^1^ | | Criteria for depression in PD | Scaner | Analysis | Software |
| --- | --- | --- | --- | --- | --- | --- | --- | --- | --- | --- | --- | --- | --- | --- | --- | --- | --- |
|  | Depression | No depression | Depression | No depression | Depression | No depression |  | Depression | No depression | Depression | No  depression | Depression | No depression |  |  |  |  |
| Andersen2015 | 10 | 18 | 55.2±7.0 | 68.4±8.2 | - | - | ON | - | - | 15.0±4.6^*^ | 4.6±3.7^*^ | 525±430.9 | 513.1±377.7 | DSM-IV | 3T | ROI | FSL |
| Ansari2018 | 40 | 19 | 57.28±7.9 | 57.5±9.38 | - | - | NR | - | - | - | - | - | - | - | 3T | - | DSI studio |
| Cardoso2009 | 20 | 16 | 64.8±7.9 | 62.5±6.5 | - | - | ON | 36.7±12.2 | 32.8±8.74 | 24.6±7.5^#^ | - | 1071 | 1009 | DSM-IV | 3T | SSQR | XBAM |
| Hu2014 | 20 | 39 | 58.1±7.72 | 54.7±10.5 | - | - | ON | 27.65±13.17 | 28.21±13.17 | 15.0±4.8^*^ | 6.8±3.1^*^ | - | - | DSM-V | 3T | ROI | SPM8 |
| Hu2020 | 20 | 47 | 58.05±7.72 | 57.94±6.90 | - | - | ON | 27.61±13.17 | 26.21±13.44 | 20.45±4.58 | 6.98±3.29 | 500±412 | 554±345 | DSM-V | 3T | ROI | FSL |
| Huang2015 | 21 | 21 | 55.1±13.7 | 54.8±9.2 | 27.2±2.2 | 27.3±2.6 | OFF | 57.7±22.4 | 50.8±22.4 | 15.0±4.9^*^ | 2.2±1.5^*^ | - | - | DSM-IV | 3T | ALFF | DPARSF |
| Huang2020 | 17 | 17 | 59.4±8.9 | 59.1±9.9 | 26.2±2.5 | 26.1±2.7 | NR | 44.1±12.3 | 40.5±9.2 | 15.0±4.10^*^ | 8.2±5.6^*^ | - | - | DSM-IV | 3T | ROI | DPARSF |
| Liang2016 | 16 | 20 | 63.5±9.87 | 61.0±10.46 | 29.51±0.53 | 29.24±2.21 | ON | 39.87±19.25 | 36.96±14.32 | 15.0±4.11^*^ | 8.30±5.37^*^ | 565±323.9 | 544.4±376.71 | DSM-V | 3T | ROI | DPARSF |
| Liao2020 | 33 | 60 | 56.5±8.27 | 56.95±9.88 | 26.79±2.25 | 27.07±2.45 | OFF | 18.58±12.52 | 14.80±9.90 | 15.0±4.12^*^ | 8.29±5.68* | - | - | DSM-V | 3T | VMHC | SPM12 |
| Lin2020 | 59 | 97 | 57.6±10.7 | 61.2±9.5 | 26.9±3.1 | 27.6±2.8 | OFF | 46.4±15.2 | 44.6±13.5 | 15.0±4.13^*^ | 13.9±6.7^*^ | - | - | DSM-IV | 3T | ICA | FSL |
| Lou2015 | 17 | 17 | 59.35±8.89 | 59.06±9.90 | 26.18±2.53 | 26.06±2.70 | NR | 44.06±12.34 | 40.47±9.19 | 15.0±4.14^*^ | 8.24±5.58^*^ | - | - | DSM-IV | 3T | ECM | DPARSF |
| Morgan2018 | 13 | 47 | 66.2±5.9 | 66.9±6.7 | - | - | NR | 24.5±12.1 | 21.4±10.3 | - | - | - | - | - | 3T | ROI | SPM12 |
| Qian2017 | 20 | 47 | 58.05±7.72 | 57.64±7.00 | - | - | ON | 27.65±13.17 | 26.21±13.44 | 15.0±4.15^*^ | 6.98±3.29^*^ | 500±412.4 | 553.69±345.43 | - | 3T | CEEMD | FSA/AFNI |
| Qiu2020 | 22 | 23 | 64.5±17.0 | 66.0±23.0 | 28.0±3.0 | 28.0±2.0 | ON | 39.0±17.3 | 35.0±9.0 | 15.0±4.16^*^ | 7.0±4.0^*^ | 505 | 275 | DSM-V | 3T | Graph theoretical  analysis | DPARSF |
| Timmer2017 | 19 | 22 | 58.4±5.3 | 61.1±7.6 | 28.5±1.3 | 28.6±1.2 | ON | 23.3±9.4 | 21.9±.8 | 15.0±4.17^#^ | 4.3±2.3^#^ | 527±240 | 626±277 | DSM-IV | 3T | rCBF | SPM8 |
| Wei2017 | 20 | 35 | 58.3±7.66 | 57.8±7.11 | 28.6±1.10 | 28.66±1.66 | ON | 28.95±13.14 | 27.24±13.39 | 15.0±4.18^*^ | 7.06±3.11^*^ | 512.8±361 | 474.0±375.67 | DSM-V | 3T | ICA | SPM12 |
| Wei2018 | 20 | 35 | 58.3±7.66 | 57.8±7.11 | 28.6±1.10 | 28.66±1.66 | ON | 28.95±13.14 | 27.24±13.39 | 15.0±4.19^*^ | 7.06±3.11^*^ | 512.8±361 | 474.0±375.67 | DSM-V | 3T | ROI | SPM12 |
| Zhu2019 | 21 | 47 | 58.1±7.53 | 57.7±7.03 | 28.67±1.11 | 28.60±1.72 | ON | 28.29±13.16 | 26.46±13.35 | 15.0±4.20^*^ | 6.87±3.10^*^ | 502.68±355 | 478.83±362.77 | DSM-V | 3T | VMHC | DPARSF |

UPDRS-III: Unified Parkinson's disease rating scale part III. LEDD: Levodopa equivalent daily dose. MMSE: Mini-mental state examination. HRSD: Hamilton Rating Scale for Depression. DSM-IV: Diagnostic and Statistical Manual of Mental Disorders version four. DSM-V: Diagnostic and Statistical Manual of Mental Disorders version five. ALFF: Amplitude of low frequency fluctuation. VMHC: Voxel-Mirrored Homotopic Connectivity. ICN: Intrinsic connectivity network. ECM: Eigenvector centrality mapping. CEEMD: Complementary ensemble empirical mode decomposition. FC: Functional connectivity. SSQR: Sum-of-squares ratio. rCBF: Regional cerebral blood flow. ROI: Region of interest. DPARSF: Data Processing Assistant for Resting-State fMRI. SPM: Statistical Parametric Mapping software. FSL: the FMRIB Software Library. NR: Not reported. ^1^ Values are mean ± standard deviation (SD)

**Supplementary Table 2:** Heterogeneity analysis

| Neural region | Side | MNI coordinates | | | Voxels | P-value | SDM-Z |
| --- | --- | --- | --- | --- | --- | --- | --- |
|  |  | X | Y | Z |  |  |  |
| Median cingulate/ paracingulate gyrus BA 23 | Na | -2 | -26 | 30 | 385 | 0.00063 | 2.187 |
| Supplementary motor area, BA 46 | Right | 8 | 6 | 52 | 214 | 0.00028 | 2.472 |
| Middle frontal gyrus, BA 46 | Right | 36 | 52 | 24 | 181 | 0.00013 | 2.744 |
| Cerebellum, hemispheric lobule VI, BA 19 | Left | -28 | -62 | -24 | 201 | 0.00060 | 2.213 |
| Inferior frontal gyrus, orbital part, BA 47 | Left | -28 | 36 | -8 | 56 | 0.00005 | 2.977 |
| Corpus callosum | Na | -36 | 20 | 38 | 28 | 0.00126 | 1.978 |
| Inferior frontal gyrus, opercular part | Right | 54 | 24 | 32 | 10 | 0.00151 | 1.917 |

MNI: Montreal Neurological Institute. BA: Brodmann area

**Supplementary Table 3:** Jackknife sensitivity analysis

| All studies but . . . | Right supplement motor area | Right frontal gyrus | Left frontal gyrus | Left cerebellum hemispheric lobule | Left median network, cingulum | Left fusiform gyrus | Right cingulate gyrus | Right temporal gyrus |
| --- | --- | --- | --- | --- | --- | --- | --- | --- |
| Hu (2015) | √ | √ | √ | √ |  |  |  |  |
| Luo (2013) |  |  |  |  | √ | √ |  |  |
| Sheng (2014) |  |  |  | √ |  |  | √ |  |
| Wang (2018) |  | √ | √ |  | √ |  |  | √ |
| Wang (2020) |  |  |  |  |  | √ | √ |  |
| Wen (2013) | √ | √ |  |  |  |  |  | √ |
| Total | 2 | 3 | 2 | 2 | 2 | 2 | 2 | 2 |
