# Supplementary material for: Altered Brain Activity in Depression of Parkinson’s Disease: A Meta-Analysis and Validation Study
Source: Front Aging Neurosci. 2022 Mar 23;14:806054. doi: 10.3389/fnagi.2022.806054 (PMC8984499; doi:10.3389/fnagi.2022.806054)
Supplement: Supplementary file 4 [file Data_Sheet_3.docx]

**Supplementary Notes 1**

*Criteria for head motion correction*

We calculated mean framewise displacement and maximum displacement. The former index was calculated using the published formula of Power et al^1^, as a combination of translational (x, y, z axes) and rotational (pitch, yaw, roll) scan-to-scan displacement, using six parameters obtained for each subject during the realignment steps. Maximum displacement was defined as the maximum absolute translation of each volume compared to the reference volume in x, y and z directions. We discarded acquisitions if they had mean framewise displacement values >0.2 mm, or if maximum displacement was greater than one voxel size.

**Supplementary Notes 2**

*Multiscale entropy (MSE)*

MSE was used to quantify the complexity of each resting-state BOLD time series, and we focused our analysis on time scales 1-10 by “coarse-graining” the pre-processed BOLD time series^2,3^. Specifically, for each time scale, the values were averaged across non-overlapping windows of different length. For example, scale 1 was the original time series (390 points), and scale 2 was derived from the average of every 2 points in the original time series (195 points). The sample entropy of each time scale was then estimated. Sample entropy is defined as the negative natural logarithm of the conditional probability that a time-series, having repeated itself within a tolerance r for m points (defined pattern length), will also repeat itself for m + 1 points without self-matches^2,3^. We chose m = 1 and r = 0.35 for this analysis following the suggestions in previous studies^4,5^.

The MSE of each voxel-based BOLD time series was then determined by plotting the sample entropy at each time scale and averaging the entropy across scale 1 to 10. Greater averaged entropies reflected greater complexity. Then the average *ROI complexity* (as defined by the averaged complexity index of the voxels within each of the three ROIs) were used in the following analysis.

**Reference.**

1 Power, J. D., Mitra, A., Laumann, T. O., Snyder, A. Z., Schlaggar, B. L. & Petersen, S. E. (2014). Methods to detect, characterize, and remove motion artifact in resting state fMRI. *NeuroImage* 84 320-341. doi:10.1016/j.neuroimage.2013.08.048.

2 Costa, M., Goldberger, A. L. & Peng, C. K. (2005). Multiscale entropy analysis of biological signals. *Physical review. E, Statistical, nonlinear, and soft matter physics* 71 021906. doi:10.1103/PhysRevE.71.021906.

3 Costa, M., Goldberger, A. L. & Peng, C. K. (2002). Multiscale entropy analysis of complex physiologic time series. *Physical review letters* 89 068102. doi:10.1103/PhysRevLett.89.068102.

4 Yang, A. C., Huang, C. C., Yeh, H. L., Liu, M. E., Hong, C. J., Tu, P. C. *et al.* (2013). Complexity of spontaneous BOLD activity in default mode network is correlated with cognitive function in normal male elderly: a multiscale entropy analysis. *Neurobiology of aging* 34 428-438. doi:10.1016/j.neurobiolaging.2012.05.004.

5 Lindquist, M. A., Geuter, S., Wager, T. D. & Caffo, B. S. (2019). Modular preprocessing pipelines can reintroduce artifacts into fMRI data. *Human brain mapping* 40 2358-2376. doi:10.1002/hbm.24528.
